# Examining Barriers to Medication Adherence and Retention in Care among Women Living with HIV in the Face of Homelessness and Unstable Housing

**DOI:** 10.3390/ijerph191811484

**Published:** 2022-09-13

**Authors:** Sofia B. Fernandez, Cindy Lopez, Cynthia Ibarra, Diana M. Sheehan, Robert A. Ladner, Mary Jo Trepka

**Affiliations:** 1 School of Social Work, Robert Stempel College of Public Health and Social Work, Florida International University, Miami, FL 33199, USA; 2 Research Center in Minority Institutions (RCMI), Florida International University, Miami, FL 33199, USA; 3 Department of Epidemiology, Robert Stempel College of Public Health and Social Work, Florida International University, Miami, FL 33199, USA; 4 Center for Substance Use and HIV/AIDS Research on Latinos in the United States (C-SALUD), Florida International University, Miami, FL 33199, USA; 5 Behavioral Science Research Corp., Coral Gables, FL 33134, USA

**Keywords:** women living with HIV, housing instability, homelessness, medication adherence, retention in care

## Abstract

Despite advances in biomedical treatments, women living with HIV (WLH) who experience homelessness and housing instability suffer suboptimal HIV outcomes, even when linked to treatment. The purpose of this study was to explore experiences of housing instability among WLH and to understand its role in their ability to adhere to antiretroviral medication and remain retained in care. Sixteen women who were linked to Ryan White Program HIV care in South Florida participated in in-depth interviews. The findings focus around four larger themes: difficulty storing medication, privacy- and stigma-related issues, inconsistent access to medication and health care disruptions, and competing and unmet physical and mental health needs. Findings underscore the importance of strategies that are responsive to the disruption of routines and are sensitive to privacy issues in shared dwelling spaces; the proactive inquiry of behavioral and environmental considerations when prescribing antiretroviral medication; and the identification and treatment of comorbid conditions. This study provides evidence for strategies to facilitate self-management and improve modifiable system realities to augment larger-level policy and funding shifts that are critically needed to end the epidemic among vulnerable populations living with HIV.

## 1. Introduction

People living with HIV who experience unstable housing conditions have poorer HIV care outcomes than those in stable housing [1]. While homelessness is the most extreme form of housing instability and includes sleeping on the streets or in other areas not meant for human habitation [2], housing instability, in general, is more widespread in the United States and encompasses a number of housing-related challenges, including living in overcrowded conditions, moving frequently, being at risk of eviction, or spending the bulk of household income on housing, all of which can impact one’s ability to maintain health [3,4,5]. In the US, women living with HIV (WLH) disproportionately belong to racial and ethnic minority groups, experience poverty, have low health literacy, and lack access to quality care [6,7]. Moreover, women living in poverty are often likely to experience less visible forms of homelessness and instability such as doubling up with friends or moving frequently [6]. For WLH who are low-income or experience homelessness and housing instability, compounded structural, socioeconomic, and social barriers present as challenges that make it difficult to benefit from available HIV care [8].

Staying retained in care and adhering to antiretroviral therapy (ART) is critical to achieving viral suppression, which is the goal of the HIV care continuum (CDC) [9]. Viral suppression, defined as having less than 200 copies of HIV/mL of blood, is critical to prevent drug resistance, slow an individual’s disease progression, and reduce the risk of further community transmission [9,10], contributing to the strategy of treatment as prevention. It is well established that homelessness is associated with non-viral suppression and an increased risk of falling out of care [11,12]. For WLH in the face of homelessness, prevention and treatment challenges that contribute to disparities along the HIV care continuum include, but are not limited to, poverty, stigma, care taking responsibilities, living in unsafe environments, a mistrust of health care systems, and lacking access to relevant HIV services and interventions that address unique and complex needs while living in homeless and unstable situations [6,7,13].

In the United States, the Ryan White Program provides services to low-income, uninsured people living with HIV. HIV services funded by the Ryan White Part A Program, which include HIV medical care, access to medications, medical case management, and ancillary supportive services, are available to persons who have a gross household income of up to 400% of the federal poverty level (FPL). Nationally, women make up 25.9% of Ryan White Program recipients [14]. Approximately 70% of women who are Ryan White Program recipients live at or below a household income of 100% of the FPL, compared to 57.4% of men in the program. Women in the Ryan White Program primarily belong to racial and ethnic minority groups, particularly African American/Black (34.2%) and Hispanic (21.5%) women [14]. Those with unstable housing have disparately lower levels of viral suppression. For example, in 2020, among Black/African American women, those with unstable housing had the lowest percentages of viral suppression [14]. Despite the availability of such services, there are still obstacles to successful adherence to ART and retention in care for many WLH in unstable housing situations.

Data collection for this study took place during the COVID-19 pandemic, a time when existing health disparities for unstably housed individuals were exacerbated, and a time when the need for linkages to permanent affordable housing was increasingly visible as a public health priority [15]. Similar to HIV, COVID-19 profoundly and disproportionately affects racial minority communities and those living in poverty, and also has a greater impact on those who are immunocompromised [16,17]. For many people, including those living with HIV, disruptions in jobs and unemployment rose due to the precautions around COVID-19 exposure [18]. Instances of homelessness and instability are expected to increase, particularly for those who are already at existing risk of experiencing housing instability [19]. Because WLH who experience homelessness and housing instability are already less likely to benefit from biomedical advancements in treatment such as ART and are less likely to achieve viral suppression compared to those in stable housing, despite having access to treatment [7,12], it important that attention is placed on efforts that support maintenance in care so that existing gaps in care and treatment are not further widened.

The purpose of this study was to explore the relationship between experiences of housing instability and the ability to manage ART adherence and remain engaged in care, from the perspective of a group of racially and ethnically diverse WLH in Miami-Dade County, FL. To strengthen the understanding of the lived realities of women in these situations, this study sought to understand subjective perspectives on the role of housing instability, giving particular attention to potential sociocultural- and stigma-related factors, and competing demands that impact daily ART adherence and engagement in care. Guided by a social ecological framework [20], which proposes that women’s experiences are influenced by multi-level factors including intrapersonal, social, and structural influences, we included questions that explored various influences to guide our understanding of experiences and to elucidate areas for intervention that can potentially improve outcomes along the HIV care continuum.

## 2. Materials and Methods

We designed a qualitative study, guided by a phenomenological approach, to address our research objective. Phenomenological studies are used to describe the shared experiences around a phenomenon for a group of individuals (typically between 5 and 25 people) [21]. Thus, we used this qualitative approach to guide our understanding of the shared, yet personal experiences of how WLH managed adherence to ART and retention in care while living in unstable housing situations. In line with the phenomenological approach to qualitative inquiry, the study was used as a method to illuminate subjective experiences and was designed to explore both intrapersonal and interpersonal participant experiences tied with housing instability and adherence to ART and engagement in care.

### 2.1. Study Location and Participants

We employed non-probability sampling to enroll women into this study. Women were recruited and enrolled between August 2020 and March 2021. Recruitment flyers were widely disseminated via email and telephone calls to Ryan White Program medical case managers working with low-income WLH in South Florida, as well as targeted listservs of Ryan White Program medical case management subrecipient agencies. In the recruitment process, case managers were asked to distribute the study flyer to potentially eligible clients. If they were interested, the potential respondents were then instructed to contact study personnel via telephone to be screened for eligibility and enrolled in the study.

To be eligible, individuals were required to be: (1) 18 years of age or older, (2) self-identified as a woman, (3) living with HIV, (4) able to provide consent to participate, and (5) have self-reported experiences of homelessness or housing instability during the previous 12 months. To determine housing eligibility, we used criteria that was adapted from the Housing Status Assessment Tool utilized by the Department of Health and Human Services [22]. Individuals were considered eligible if they responded “yes” to any of the following conditions: Over the past 12 months, have you or are you currently: (1) staying in a shelter, transitional housing, or other non-permanent location, (2) at risk of losing housing due to inability to pay rent, being asked to leave, or eviction, or (3) at risk of losing housing or needing to leave current housing because of safety concerns, host family/friend risk, or conflict? While we were particularly interested in the experiences of racial and ethnic minority women, women from all races and ethnicities were eligible to participate if they were fluent in either English or Spanish, as these were the two language options for conducting the interview.

### 2.2. Interview Protocol and Procedures

Interviews were conducted via an audio recorded telephone call due to COVID-19 social distancing requirements. Study staff described the purpose of the study as a research study that would be conducted as a phone conversation to understand how women in unstable housing or homeless situations were able to manage their HIV care. If an individual was interested and eligible, research staff arranged with the participant to have a 1-h phone call at a time that was convenient for the participant. Participants were asked to identify a time when they would have 1 h of privacy to maximize participant protection and minimize potential risk of loss of confidentiality. This was stressed during the informed consent process, given that interviews were conducted remotely and not at a private facility. Study staff emailed an electronic consent form to eligible participants before the interview meeting day. Prior to conducting the interview, study staff read the informed consent form with the participant and assessed that the participant understood the procedures, potential risks and benefits, and voluntary nature of participating in the study. After participants provided verbal and electronic agreement through the online consent form, the study staff proceeded with enrolling the participant in the study and began the interview.

Interviews were conducted using a semi-structured interview guide that was developed by reviewing the literature and with input from the study team. Interview questions were designed to gain a deeper understanding of the relationship between housing situations and the participant’s ability to adhere to ART and remain engaged in care. In line with a social ecological framework, the interview guide included broad questions about challenges around adherence and retention and probes around interpersonal and social influences. A brief set of demographic and background questions were also collected prior to starting the interview. In line with a qualitative approach, interview topics covered in each interview varied slightly based on the participant’s unique experiences, but general prompts and questions were used to guide all interview discussions. Table 1 displays a list of interview protocol prompts that were used during data collection. Participants received a USD 60.00 Amazon gift card for their participation in this study. All study procedures were approved by the University’s Institutional Review Board, and all participants provided electronic informed consent before participating in this study.

### 2.3. Analysis

Each interview was audio recorded, transcribed verbatim, and de-identified for subsequent analysis. To ensure accuracy of the data, the research team selected parts of each interview transcript to verify equivalence with the audio file. The qualitative data were then analyzed using techniques of thematic analysis and an iterative coding process with the goal of developing themes around shared participant experiences [23]. First, the primary researcher read through the data (e.g., transcripts) for each of the interviews and highlighted significant statements around the research questions to create a preliminary codebook [23]. Two coders tested the preliminary codebook by coding two interviews independently, adding descriptive codes as needed. After meeting and finalizing an initial codebook structure, all remaining transcripts were coded independently, using an iterative process, where coders met to compare assigned codes and assess interpretations of the transcripts after coding each interview. Descriptive codes were added as analysis occurred through a consensus process. Coders discussed their perspectives on participant experiences around adherence and retention challenges, assessed their inter-rater agreement on the application of codes, and discussed emerging codes. Meetings were used as a tool to aid in reflectivity and reliability of the analysis by conducting the coding through a collaborative process. Themes were developed from the descriptive codes to capture shared experiences or larger themes [23]. Representative, verbatim quotes were then selected to illustrate key findings. NVivo 12 software was used to store, code, and organize our data analysis.

## 3. Results

Sixteen women (31% Hispanic, 69% Non-Hispanic Black; mean = 48 years old; standard deviation: 8.78 years) participated in this study. One individual who was eligible but who could not provide electronic informed consent was unable to participate in the study. Women reported their housing situations over the past 12 months, which included literal homelessness (i.e., sleeping on the street and staying at a shelter), as well as doubling up with others, staying in subsidized rental housing, and staying in residential sober living facilities. Almost half of the participants (43.8%) were currently receiving some form of housing assistance. Most participants reported several housing situations over the course of the prior 12 months, reflecting the transient and cyclical nature of unstable housing and homeless situations. Table 2 identifies participant’s housing situation at the time of the interview and the length of time spent at that current location.

Qualitative results focus on participant perspectives of their ability to adhere to ART and remain engaged in their care while experiencing homelessness and/or housing instability. The majority of the transcriptions elucidated challenges around adherence and less specific descriptive information was provided around retention in care. In sum, we focus on four larger themes that described barriers to adherence and retention among participants: (1) difficultly storing and misplacing medication, (2) privacy- and stigma-related issues, (3) inconsistent access to medication and health care disruptions and, (4) competing physical and mental health issues. Table 3 provides additional illustrative quotes.

### 3.1. Storing and Misplacing Medication

Across different housing situations, transience and instability of housing situations impacted the ability to take medication as prescribed and stay engaged in care. For many participants, barriers included difficulty storing medications, which was required to effectively take medication as prescribed daily. Experiences included misplacing medications after attempting to hide medications in non-personal spaces as well as having medication stolen. Most women reported taking their medication as best as possible but faced substantial challenges related to safely storing and transporting medication while experiencing unstable housing.

*There have been times where I have lost it because I do keep it hidden. Which is, actually, what I’m experiencing now…Yes I do [keep my medication hidden] …I don’t leave it in my pocket for whatever reason… Probably because I feel like if someone uses my bathroom. You know, a lot of people that use medication, do know what they are for. And that’s not for everyone to know depending on the knowledge of the person and what they know. I just don’t leave it around. So, sometimes that results in me misplacing… A lot of times people just steal this stuff because a lot of times, as I understand it, some of these medications, people will buy them on the streets… I think that’s more of a personal issue with me that I have to deal with. Because I know that that’s just my issue and that’s just what I do. But, um, not everybody necessarily suffers from that.*

US born, non-Hispanic Black, 43-year-old.

Participants described ways of storing or keeping their medication on them despite having transient and changing living situations. This included hiding medication in public spaces such as in bushes and also using various strategies to store medication such as in cooler bags:

*I had to keep them in a cooler, you know, in a cooler bag. I had to keep my meds in there. I bought it from a Dollar Tree Store.*

US born, non-Hispanic Black, 37-year-old.

Participants described difficulty taking medication due to having belongings in several places and having disruptions in daily routine. For example, one participant described:

*Sometimes I would miss it because I was staying over somewhere, and my things were somewhere else.*

US born, White Hispanic, 56-year-old.

Lack of ability to safely store and retain medication was consistently folded into the experience of housing instability, which included constantly moving, having personal items in various and non-personal locations, not having resources needed to store medication, and having daily routines that were inconsistent, making it hard for daily adherence regimens.

### 3.2. Privacy Issues with Those with Whom They Live

Privacy issues repeatedly came up for women, especially those who were living in shelters/housing facilities, in crowded environments, or living with people they did not trust. These experiences impacted their ability to adhere to ART and remain engaged in care because of issues around participants wanting to protect or “hide” their status. Several women who were living with others often described conflict and distrust with household members that impacted their ability to take their medication.

*I have to be even more cautious who I share with. I mean I even have a very close friend that doesn’t even know. She probably suspects, but I’ve never shared with her because, next time you have a friend, you should be careful who you tell. Because some people they seem very educated, they seem like they know a lot of things. The moment you tell them, they’re scared! So generally, I’m not going drink, take my drugs openly. Or like in a family dinner, I have my drugs. I’ll make sure, I’ll make time to take my drugs. I’ll go to the room. I’ll be in my space, take the drugs. But I will not be open about it or keep the drugs… At the [Shelter], I request them, I always take the time to just see what is in there. It just, living this part of your life where you have to be extra cautious, keeping the information to yourself. I have learned to be cautious.*

African born, non-Hispanic, Black, 40-year-old.

Other participants described situations where they did not have privacy, which resulted in skipping doses of medication and “doubling up” on medication to avoid others witnessing them taking their medication:

*Sometimes people would be around, so sometimes I would take it in the morning, sometimes I would take it at night or sometimes I would take it early, early morning. It varied. I can’t tell you my exact schedule but it would all vary when nobody was around, that’s when I would take it. And I know you’re not supposed to double up, but if I knew I was going to be around somebody all day I would take two of my medicines the day before*.

US born, Non-Hispanic, White, 36-year-old.

Privacy and stigma issues impacted not only taking medication as prescribed, but also making it to doctor appointments. Some women described difficulty maintaining doctor visits to remain in care due to the frequency of visits and its potential impact on social relationships.

*It’s such a task to go to a doctor’s visit, try to get some rest, and then make it down to [location of doctor which is far from current location of participant] to get the medicine. So, if I was living with someone, and they see that on a day off I have all of these extra-curricular to do, then people will start wanting to wonder, ‘Why does she go to the doctor so often? What kind of medication is she gonna pick up every month at this place?’*

Jamaican born, non-Hispanic Black, age not provided.

Stigma and privacy manifested as themes that were pervasive to women’s experience and were particularly present in the context of sharing living spaces with others who did not know a woman’s HIV status.

### 3.3. Inconsistent Access to Medication and Health Care Disruptions

Barriers affecting taking medication as prescribed also included a lack of consistent access to medication and health service disruptions. Women described this inconsistent access to medication and health care service disruption as a result of falling out of care due to a lack of compliance. This was largely attributed to women missing required applications, lab tests, and system requirements to remain active in care and receive medication.

*What that looked like was missed application for insurance. Having a gap in medication. So that’s what that would look like and, only by the grace of God, I remain virally suppressed. But if you can’t get your medication, for whatever reason, you can’t take it and that is a hindrance to your health care and your well-being. Cause the whole month when I didn’t have it it’s like, ‘Oh my God, I don’t know what my viral load is. I don’t know what my CD4 is’. You don’t know what’s going on with your health because everything is done through labs. So those were some stressful…I mean they may have told me by email to change but I didn’t reapply. And when you don’t reapply, everything gets shut off, everything. Your food stamps, your Medicaid. Everything gets cut off until you reapply.*

US born, non-Hispanic, Black, 50-year-old.

Another participant said:

*And then of course with the care being denied, or the care being trampled. That’s also been a thing about me having to go and restart and get all of this set up, set up a time with the case manager and then make an appointment to see the doctor. And then go to the doctor, and then, go to the lab. You know, that also causes issues with that as well… So, that has sometimes caused me to be out of care.*

US born, Non-Hispanic, Black, 43-year-old.

Another participant explained:

*I did have an incident where I wasn’t able to take my medicine for 7 days ‘cause I wasn’t aware that my program had expired. I got no letter, no nothing from the case managers. I wrote corporate, I asked, I explained. I heard nothing from them yet.*

US Born, non-Hispanic, Black, 50-year-old.

System requirements were often perceived as too burdensome, unfair, and unrealistic to be met by women in unstable housing experiences. Women described their access to medication and care as full of “barriers” and their care being “trampled” on when they were not able to meet requirements for care renewal. This resulted in them falling out of compliance and having to restart application processes.

### 3.4. Competing Physical and Mental Health Needs

Women with housing instability also reported unmet and competing needs that included depression, chronic stress, substance use issues, competing physical illnesses such as pancreatitis, and a lack of food. These other concerns were described as barriers to adherence to ART and retention in care. Many women reported having comorbid conditions that also required medication and care.

*Sometimes I feel very depressed, I feel down, and I don’t feel like doing anything, sometimes I feel like ‘Oh, I’m tired of this. I’m tired of taking medicine. I’m gonna give up and I’m gonna let it slide and see what happens... And I’ll be like, ‘I’m gonna die anyways someday.’ You know sometimes people think that negative stuff, trying to hurt your own self and I do that sometimes on myself because I’m human. I feel. But I still continue, you know, going forward because of my staff like I said before and my mom. They are my only family. That’s it.*

Nicaragua Born, Hispanic, White, 43-year-old.

Another participant described:

*I mean stress trying to find a place, stress, stress, plus, you know me. I can honestly say that for me and 20 years being positive and taking medication for all these years, I’ve had those times where I’m like I don’t wanna take this pill, I wanna skip a day, I wanna skip a week. I’m going to skip a month because of the day to day living situations that become overwhelming at times. And I think I’ve never had a problem of taking it in these 20 years but at times it’s like you don’t really care. But then you do remember that you have to care. So I couldn’t, I wouldn’t sit here and tell you that, yeah I’m an advocate, I teach people, I tell people this and that and I’m perfect. Which would be a lie. Cause sometimes I look at these big ass fat horse pills and I’m like, I’m not taking those pills.*

US Born, Non-Hispanic Black 50-year-old.

Several participants described how drug use played a role in their engagement in care and medication adherence while homeless:

*Anytime when I begin to use, I quit taking my medications. That’s the first thing to go out the window, it’s my medications and going to doctor’s appointments. Cause I have no time for it. I only have time for drinking and drugging or using. And being homeless, you can’t eat on time or take medications on time, or at least I couldn’t. You know? So, yeah, every time that I become homeless, I quit taking medications and been out of care for a while. I’m very lucky that I respond well to medications. They haven’t put me on anything that I haven’t responded well to and I guess I’ve been on 5 different regimens because they won’t put you back on the one that you were on once you stop taking it. So um, but I’m undetectable. And I become undetectable quickly once I get back into care again.*

Non-Hispanic White, 55-year-old.

Competing and unmet needs also included a lack of food to take with their medication:

*I was taking my medicine without food sometimes because I couldn’t afford the food so sometimes I would take the medicine and it would make me feel so horrible.*

Jamaican Born, non-Hispanic Black, age not provided.

Another participant stated:

*Somedays when I was on the beach and I didn’t have no money, no nothing, no food, no nothing, oh my God, it was hard. Cause that medication, you have to take with food, or it will affect your body. So, I was like crazy. Sometimes I ate out of garbage can... I begged, I panhandle. I used to prostitute and, then, that stuff got old. I tried selling my body for food. Sometimes it was just hard.*

US Born, non-Hispanic, Black, 37-year-old.

The competing physical and mental health needs were described as exacerbated by or as a result of experiencing unstable housing. Competing needs were pervasive enough (e.g., stress, depression, lack of food, and drug use) that they were reported to prevent women from managing care. Stories highlighted the interconnectedness of housing instability and homelessness on daily functioning, including mental and physical health abilities needed to adhere to medication.

## 4. Discussion

This study sought to examine experiences of housing instability and the ways in which housing instability impacted ART adherence and retention in care from the perspective of WLH who experience unstable housing. Findings underscore that housing situations, stability, and relationships play a role in women’s abilities to stay adherent to medication and remain engaged in care. Difficulty storing medication, disruption in routines, inconsistent access to medication and health care disruptions, privacy issues with persons with whom they live, and competing physical and mental health concerns among women living in unstable housing situations presented as significant barriers to adherence and retention in care.

Research has clearly documented that homeless experiences result in lower rates of viral suppression and poor HIV care outcomes, but the lack of evidence about specific barriers to ART adherence and retention in care and the influence of social and structural factors have made it hard to create appropriate interventions [24]. Our findings identify specific areas needed for targeted interventions that may help to mitigate challenges in this population. Together, these noted obstacles present as challenges that complicate adherence to ART and retention in the health care system among WLH and underscore the need for specialized and tailored interventions to prevent disengagement, improve adherence, and ultimately improve viral suppression rates among this population.

Our findings suggest that strategies that are responsive to the changing and transient nature of housing situations, which include consideration of the lack of daily routines, and those that are sensitive to privacy issues when living with others are needed. In other HIV populations, routines and stability have been identified as predictors of medication adherence. Research suggests that the extent to which one’s daily life is structured and routinized is an important factor in understanding medication adherence [25]. This becomes increasingly difficult in unstably housed populations. Light-weight storage containers as well as clocks, watches, or other types of daily/physical reminders can be useful given the lack of stable routine when experiencing homelessness, which is critical for successful adherence. Low-cost and pragmatic interventions such as small pillboxes remain a simple intervention that can have an impact [26]. Furthermore, given that the women were able to access cellphones during this study and one participant described the potential for text message reminders, mobile phone reminders for women who are unstably housed (e.g., for appointments, medication pick-ups, and adherence) may be a potential method of intervention. Other studies have shown that homeless populations have cellphone use that matches the general population, suggesting this as a potential platform for SMS or other mobile interventions that are tailored for the population [27].

Our findings shed light on the realities of women in this study who identified ways of storing medications, such as in small coolers and in outdoor areas (e.g., bushes). Although seemingly resourceful, some of the modifications identified are potentially harmful, for example, when medications need to be stored at cool temperatures. This can be increasingly dangerous in warmer climates such as in South Florida. Furthermore, as a result of inconsistent daily routines, women reported doubling up on medications, another problematic modification that is not recommended for those prescribed ART [28], due to the potential consequences of increased medication doses on the liver, kidneys, or an interaction with other medications. Additionally, women described a lack of food. These behavioral and contextual considerations of adherence highlight the need for the proactive inquiry of client’s environmental conditions, as these considerations can impact the medication chosen and can allow providers to assist in addressing client needs.

Moreover, given the noted barriers and realities of women who are in unstably housed stations and who may have a limited ability to openly take, appropriately store, and adhere to medication daily, there is a potential utility for injectable ART for this population. Unstably housed women may be good candidates for long-acting injectable ART regimens [29]. Contrary to the daily requirement of common ART regimens, long-acting injectables would require going to the clinic to receive an injection once or every two months. Long-acting injectable treatments may be particularly useful for people from unstably housed populations who are able to attend clinic appointments but struggle with a daily adherence to pills. However, cabotegravir and rilpivirine, two current long-acting injectables, are only approved for individuals who have undetectable viral loads, and they are not yet approved for people who struggle with adherence [29]. Critical consideration is required given that drug resistance could develop if an individual stops the injectable medication. Current clinical trials are underway to investigate long-acting injectables for individuals with poor adherence [30], which can inform future implementation with this population. Future studies would also need to consider the perspectives of women about potential acceptability of long-acting ART, including an exploration of the ability to travel to the clinic once or every two months. Outreach or mobile services maybe particularly useful in these circumstances.

Beyond daily adherence issues, system requirements were difficult to meet for these women causing barriers to retention in care. Women described an inability to access medication because of falling out of care, specifically during instances when participants were not compliant with the system requirements. Throughout the COVID-19 pandemic, federal and community agencies amended several compliance requirements including removing the requirement for viral load lab testing and in-person visits [31]. Challenges with transportation, making it to the clinic for appointment times, and clinic stigma have been identified as significant reasons for a lack of retention among low-income HIV populations in previous research, even before COVID-19 social distancing requirements [32]. COVID-19-related modifications such as more flexible requirements for staying in care (e.g., not requiring viral load lab tests every 6 months) could potentially benefit unstably housed WLH who may find it hard to adhere to requirements for remaining compliant. Still, the responses to COVID presumed housing stability (e.g., sending medications via mail-order), and did not address issues of where to keep medications without having access to a refrigerator. COVID-19-related system modifications need to be monitored for the impact on HIV patient outcomes.

While stigma and privacy issues were presented as a distinct theme, it is important to note that issues of stigma and privacy were intertwined throughout almost all of the themes, highlighting the salience and importance of this issue. For example, many of the stories around storing medication involved some layer of stigma or privacy concern. Women described keeping medication hidden and also skipping doses because they were around others. This suggests that relevant and responsive interventions will need to carefully incorporate the intricacies of disclosure and privacy among WLH and will need to address the social realities of living in homelessness and unstable housing, as such structural and social influences have an impact on a woman’s health and well-being [33,34].

While participants had a wide range of housing situations, almost half were currently receiving housing assistance. Other studies have found that access to housing assistance and stable housing that is subsidized to an affordable level is associated with better adherence and reduced risk behaviors in people living with HIV [35]. Programs such as Housing Opportunities for Persons with AIDS (HOPWA) provide housing assistance to low-income people living with HIV, but its resources are limited, and more assistance is needed [31].

Additionally, just as housing stability is needed to improve the adherence to HIV treatment [35], appropriate identification and treatment of underlying mental and physical health conditions, including substance use, will be necessary to achieve emotional and physical wellness that contributes to ART adherence and improved treatment outcomes. Clinical guidelines for treating unstably housed individuals with HIV stress the importance of treating co-occurring or comorbid conditions such as mental health and substance use disorders, simultaneously, in coordinated care [36]. This underscores the need for patient-centered care strategies that treat patients for multiple health challenges in an integrated manner.

## 5. Limitations

This study has several limitations that should be noted. First, this study represents the perspectives of a small sample of women who were recruited from HIV medical case management agencies and thus were currently engaged in HIV care. In addition, it is possible that social desirability bias impacted participant responses and led to an underreporting of non-adherence or other potentially stigmatized behaviors. To mitigate this potential bias, we created culturally sensitive interviews with an interviewer who had training and experience in culturally sensitive data collection with HIV populations. Finally, the women who participated may represent a group of women who were more involved in their care and were currently in a stable part of their housing experience, leaving out the perspectives of those who were potentially experiencing more or different challenges. Though we sought to recruit 25 women in this study, there were several challenges to recruiting this population during the context of the pandemic. Nonetheless, the women who participated in this study provided valuable and salient insights that can drive our efforts to serve this population.

## 6. Conclusions

To achieve the goals outlined in Ending the HIV Epidemic: A Plan for America [37], there remains a challenge to improve viral suppression rates among people living with HIV, especially among those in geographic hotspots and among groups with a disproportionate burden of poor HIV outcomes. This study critically examined experiences of housing instability and the ways in which unstable housing situations impact the adherence to ART and the retention in care. Significant barriers to HIV adherence and retention included storing and misplacing medication, inconsistent access to medication and health care disruptions, privacy- and stigma-related issues, and competing physical and mental health issues. These findings augment our understanding of the unique experiences of unstably housed populations and can be used to improve the development of adherence interventions and services for low-income WLH, specifically those who face housing instability. Understanding the roles of housing instability as a structural determinant in the HIV epidemic among women is particularly important because these realities prevent women from getting the HIV care and treatment they need. While, taken together, our findings, underscore the need for housing as a public health strategy that can improve HIV care outcomes, this study provides evidence for strategies that can help facilitate self-management and improve modifiable system realities to augment larger-level policies and funding shifts that are critically needed to end the epidemic.

## Figures and Tables

**Table 1:** Sample Questions Included in the Interview Guide.

Please describe to me more about your living situations over the past year. Please tell me more about who you have lived with over the past year. Describe the relationship you’ve had with that person(s). Tell me about any care taking and household responsibilities. Please tell me what has made it difficult for you to maintain housing now or in the past. Please tell me about your experience with disclosure of your HIV status to your household or family members. Please tell me about how your living situation affects the decisions you make regarding your health, if at all? Please tell me about how your living situation affects your ability to maintain your HIV care, if at all? Please describe a typical day for me and specifically tell me about how you manage taking your HIV medication. Tell me about a time that you weren’t able to take your HIV medication as prescribed or a time you weren’t able to make it to your doctor appointment. What supports do you need to help you better adhere to your medication regime and improve your health?

**Table 2:** Demographic and housing characteristics of participants (*n* = 16).

	No. (%)
Age, mean (SD)	48.47 (8.78)
Where were you born, no. (%)	
US	12 (75.0)
Puerto Rico	1 (6.3)
Nicaragua	1 (6.3)
Jamaica	1 (6.3)
Africa	1 (6.3)
Ethnicity	
Hispanic/Latino	5 (31.3)
Non-Hispanic/Latino	11 (68.8)
Race	
Black/African American	11 (68.8)
White	4 (25.0)
Prefer not to answer	1 (6.3)
Marital status	
Single	7 (43.8)
Married/living together with a partner	2 (12.5)
Separated or divorced	5 (31.3)
Widowed	1 (6.3)
Engaged	1 (6.3)
Current living situation	
Own house/apartment	7 (43.8)
Parent’s house/apartment	1 (6.3)
Someone else’s house/apartment	4 (25.0)
Residential drug, alcohol treatment facility, or halfway house	2 (12.5)
Single room occupancy (SRO)	1 (6.7)
Shelter	1 (6.7)
How long have you stayed at the place you stayed last night?	
Less than a week	1 (6.3)
More than 1 week but less than 1 month	1 (6.3)
1–3 months	4 (25.0)
More than 3 months but less than 6 months	9 (56.3)
6 months or more	1 (6.3)
Who do you currently live with?	
Alone	2 (12.5)
Spouse/partner/significant other	4 (25.0)
Other adult family members/relatives	1 (6.3)
Dependent children/grandchildren	3 (18.8)
Friends/roommates/other unrelated persons	4 (25.0)
Group home/shelter	1 (6.3)
Other	1 (6.3)
Care for children under 18 years old	
Yes	5 (31.3)
No	11 (68.8)
Highest level of education	
Grades 1 through 8	3 (18.8)
Grades 9 through 12	3 (18.8)
Grade 12 or GED	3 (18.8)
Some college, associate’s degree. or technical degree	5 (31.3)
Bachelor’s degree	2 (12.5)
Receive housing assistance	
Yes	7 (43.8)
No	9 (56.3)

**Table 3:** Identified barriers for medication adherence and retention in care for women living in unstable housing.

Theme	Illustrative Quote(s)
Storing and misplacing mediation	I wouldn’t keep it [HIV medication] on me. I would hide it in the bushes. I wouldn’t take it at the same time every day…But nobody would know it ‘cause I wouldn’t tell nobody I had it… Yup, so no one would see it or no one would get my purse and see…I would take the name thing off and hide it in the bushes and then, when nobody was around I would take it. US Born, non-Hispanic White, 36-year-old.
Privacy issues with persons with whom they live	I’ve not shared my status with many people that know me so I need the privacy to make sure that I be taking my medicine. Jamaican born, non-Hispanic Black, age not provided.
Inconsistent access to medication and health care disruptions	Another issue that I had was with my labs to renew my case through ADAP. The labs are… were old. And I haven’t been to the doctor. The last time that they went to the doctor, I don’t know if they made an error or what they did. But they did not give me a return appointment. They told me ‘Oh, we don’t have a return appointment for you so you just gone’. So, what ended up happening was that my labs ended up getting over the 6 months due date. So, what they were saying was for me to renew my med, for me to renew my case, I need to have current labs. In order for me to have current labs, I have to schedule an appointment with the doctor. And go down there and wait for the labs to come. That’s ridiculous…And then they don’t send any reminders as to when… Like they verbally tell you, but they don’t send me any reminders. And even though I could write it down, that’s me slipping it and know when my paperwork is due, but they don’t have a system, a text message system, where they say, ‘Hey, look your insurance is going to end up in…’ None of that. So, once it goes out, it’s a mess. Yeah, there’s a lot of barriers, I do not like that system. I don’t, I’m sorry. US born, Non-Hispanic, Black, 43-year-old.
Competing physical and mental health concerns	Last time was drugs. I wouldn’t care. I would forget. I would rather have drugs before my medication. But now it really don’t affect me. I know when I have to take my medication. Because I would stay high. I wouldn’t want to go pick up my medication. Puerto Rico Born, Hispanic, 36-year-old.

## Data Availability

The data are not publicly available in order to protect any potential identification of participants.
